# Transcriptomic insights into *Candida albicans* adaptation to an anaerobic environment

**DOI:** 10.1128/spectrum.03024-24

**Published:** 2025-05-22

**Authors:** Karen D. Zeise, John R. Erb-Downward, Gary B. Huffnagle

**Affiliations:** 1 Department of Microbiology and Immunology, University of Michigan 242912, Ann Arbor, Michigan, USA; 2 Mary H. Weiser Food Allergy Center, University of Michigan 1259, Ann Arbor, Michigan, USA; 3 Department of Molecular, Cellular, and Developmental Biology, University of Michigan 1259, Ann Arbor, Michigan, USA; 4 Division of Pulmonary and Critical Care Medicine, University of Michigan 569795, Ann Arbor, Michigan, USA; University of Wisconsin-Madison, Madison, Wisconsin, USA

**Keywords:** *Candida albicans*, anaerobic, transcriptomics

## Abstract

*Candida albicans* is a clinically significant fungal pathogen capable of adapting to diverse host environments, including steep oxygen gradients ranging from ~21% oxygen to anaerobic. The ability to withstand varied oxygen levels is paramount to establishing colonization and persisting in host niches, and oxygen deprivation can also augment antifungal resistance. In this study, we used RNA sequencing to compare the global transcriptomic profiles of two strains of *C. albicans* (SC5314 and CHN1) grown purely anaerobically to those grown aerobically. In *C. albicans* SC5314, we observed a strong induction of the alternative oxidase *AOX2* and several genes encoding subunits of mitochondrial enzyme complexes I, II, and V, signifying a shift to alternative respiration. Consistent with the diminished ATP production from this process, there was a significant downregulation of genes associated with growth and metabolism, including histones and ribosomal proteins, as well as chitinases and other genes involved in cell wall remodeling. Interestingly, the anaerobic *C. albicans* cultures had decreased expression of candidalysin (*ECE1*) and other virulence factors, contrasting with other studies reporting enhanced pathogenicity under oxygen deprivation. There were a greater number of significantly upregulated genes in *C. albicans* CHN1 compared to SC5314; however, most of the top 50 upregulated genes under anaerobic conditions were consistent between the two strains. The predominant difference in down-regulated genes between the two strains could be mapped to differences in hyphal transformation under aerobic conditions. Overall, our study provides a window into the molecular mechanisms of *C. albicans* adaptation between aerobic to anaerobic environments.

**IMPORTANCE:**

*Candida albicans* is a leading cause of fungal infections in humans, posing significant clinical challenges due to its remarkable adaptability and increasing antifungal resistance. Anaerobic environments can promote antifungal resistance, necessitating a deeper understanding of how *C. albicans* adapts to anoxia. While much research has been done to identify mechanisms underlying adaptation to hypoxia (i.e., low oxygen), this is the first study evaluating the global transcriptomic response of *C. albicans* to anoxia (no oxygen). Here, we uncover key transcriptomic changes that enable *C. albicans* to survive in the absence of oxygen, which are distinct from those identified under hypoxic conditions. Our research addresses a gap in current knowledge that may be exploited for combatting antifungal resistance.

## INTRODUCTION

As a common fungal inhabitant of the human microbiome, *Candida albicans* has evolved strategies to thrive in diverse environments with varying nutrient and iron availability, oxygen levels, pH, and microbial competition. Oxygen is a major determinant of niche suitability for microbes, with levels ranging from ambient (e.g., on the skin) to anaerobic (e.g., in biofilms or anaerobic microenvironments within the colon) (1). The gastrointestinal tract is considered to be the primary reservoir for *C. albicans* and from where most disseminated infections originate (2). As such, the ability to survive in oxygen-limited conditions is important not only for *C. albicans* colonization along the gastrointestinal tract but also for its virulence. Low oxygen (hypoxia) has been shown to trigger β-glucan masking on the *C. albicans* cell wall via a mitochondrial- and cAMP-protein kinase A (PKA)-dependent signaling pathway, enabling it to evade *in vitro* phagocytosis by innate immune cells (3, 4). However, in other contexts such as post-antibiotic recovery, oxygen may actually be required for *C. albicans* expansion, and resident anaerobic bacteria can inhibit fungal blooms by limiting oxygen availability in the gut epithelium (5). Thus, oxygen may actually be a double-edged sword when it comes to *C. albicans* colonization and pathogenesis.

In recent years, there have been a number of investigations into the metabolic and transcriptomic responses of *C. albicans* to hypoxia (6–10). Hypoxia induces significant shifts in respiration and carbohydrate utilization pathways critical for downstream hypoxic metabolic reprogramming, including increased glycolysis and fermentation, and repression of the tricarboxylic acid cycle and oxidative phosphorylation (6). Upregulation of genes associated with glycolysis is an early hypoxic response that requires the transcriptional regulators Tye7 and Gal4 (11). Reduced levels of ergosterol and other cell membrane lipids that depend on oxygen for their biogenesis have been observed following this response (6), which coincides with temporal transcriptomic analyses showing delayed activation of genes involved in ergosterol and unsaturated fatty acid biosynthesis (8), mediated by Upc2 and Efg1 (8, 9, 12). The transcription factor Efg1 is also a key regulator of *C. albicans* filamentation that is essential for infection and invasion of host tissue (13, 14).

While the strategies used by *C. albicans* to adapt to hypoxic environments have been studied extensively, little is known about how the fungus responds to completely anoxic environments such as the colon. Previous studies have shown that anoxia renders *C. albicans* more resistant to antifungals (15) and markedly affects its growth kinetics and phenotype (15–17), underscoring the need to investigate the underlying transcriptomic changes, as it could provide valuable insight into antifungal resistance mechanisms and inform the development of new antifungal therapies. Thus, the primary objective of our study was to use RNA sequencing (RNA-seq) to compare the global transcriptomic profile of *C. albicans* SC5314 cells grown anaerobically at physiological temperature (37°C) to that of cells grown aerobically under otherwise identical conditions. We used rich media in all of our experiments to facilitate fungal growth, as anoxia greatly reduces the growth rate of *C. albicans* (15, 16). Additionally, we were interested in determining whether differential gene expression in anaerobic vs aerobic conditions could be replicated in the *C. albicans* strain CHN1, which belongs to a different species clade from SC5314 and exhibits distinct colonization dynamics in mice (18–20). Our analyses revealed a strong induction of alternative respiration mediated by AOX2 and mitochondrial complexes I, II, and V in the anaerobic cultures, accompanied by widespread downregulation of genes associated with pathogenicity, metal acquisition, and metabolism. Importantly, differential gene expression in anaerobic vs aerobic cultures was similar between SC5314 and CHN1, suggesting that these two strains have evolved similar adaptation mechanisms to anoxia. These findings shed light on previously unexplored genes and pathways important for *C. albicans* survival in the absence of oxygen, paving the way for future research into strategies to combat *C. albicans* infections and antifungal resistance.

## RESULTS

### RNA sequencing of *C. albicans* SC5314 under aerobic and anaerobic conditions

**Fig 1:**
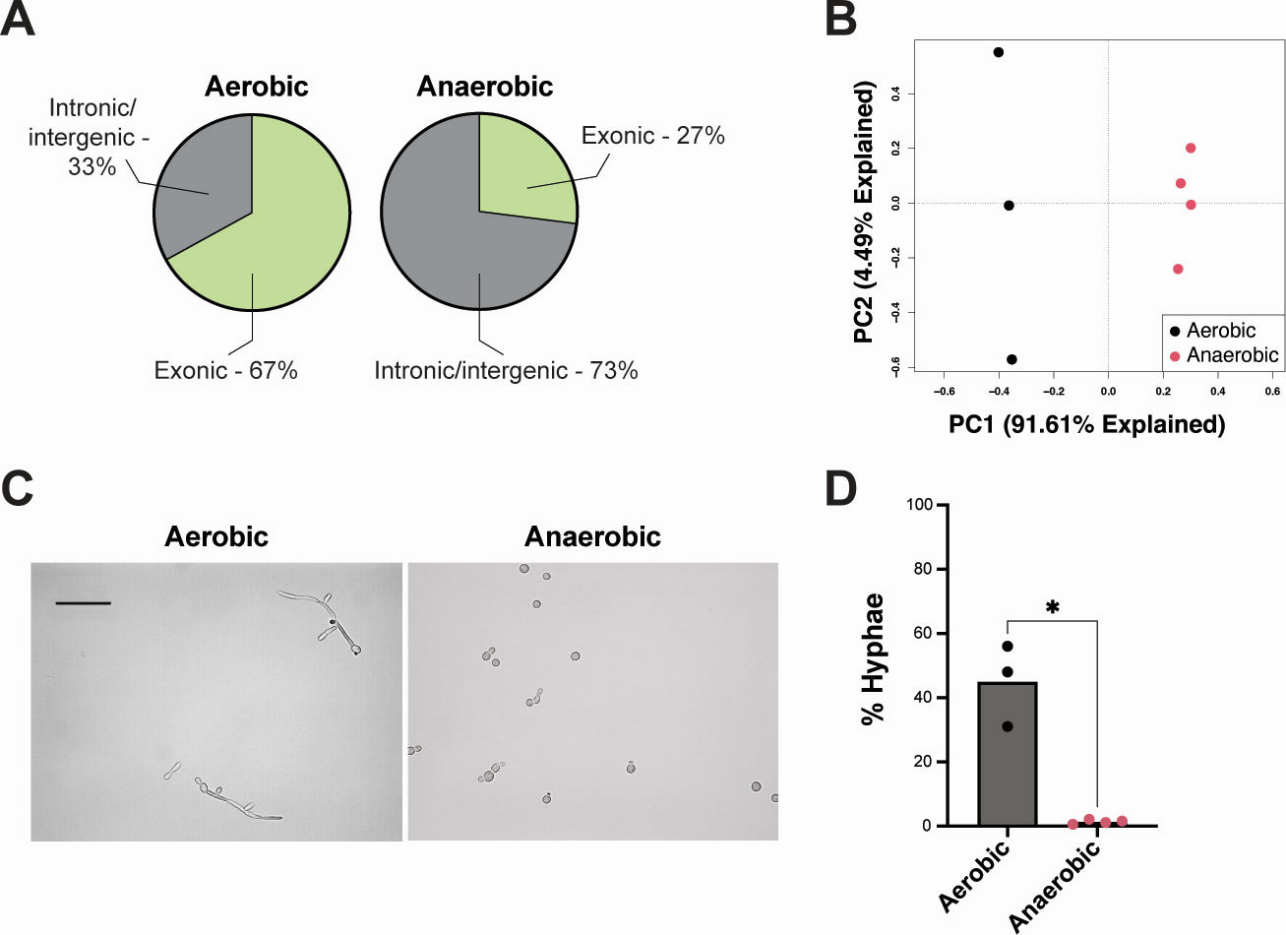
Sequencing results and phenotypic observations from *C. albicans* SC5314 cultures. (**A**) Percentages of sequenced reads that mapped to exons (light green) vs intronic/intergenic regions (gray). (**B**) PCA of the aerobic and anaerobic cultures based on their transcriptomic profiles. (**C**) Brightfield micrographs of the aerobic and anaerobic cultures at the time of collection for RNA isolation. Scale bar = 20 µm. (**D**) Quantification of *C. albicans* hyphae in the aerobic and anaerobic cultures. *P < 0.05, unpaired *t*-test.

To recapitulate the extreme oxygen gradient encountered by *C. albicans* along the gastrointestinal tract (ranging from anoxic microenvironments in the intestinal lumen to ambient oxygen in the oral cavity), we grew *C. albicans* SC5314 in a nutrient-rich media (de Man, Rogosa, and Sharpe [MRS]) under aerobic (~21% oxygen) and anaerobic (0% oxygen) conditions at physiological temperature (37°C). Total RNA was isolated and sequenced from at least three separate exponential-phase cultures per condition. For the aerobic cultures, we obtained an average of 2,287,314 reads per sample, of which >98% were successfully mapped to the SC5314 reference genome (assembly 22). For the anaerobic cultures, an average of 32,772,211 reads were sequenced per sample, and >97% of those reads could be mapped to the reference genome. Table S1 shows the raw counts obtained across all samples. Sixty-seven percent of the total reads obtained from the aerobic cultures and 27% of the reads from the anaerobic cultures mapped to exons (Fig. 1A). To visualize similarities in transcriptomic profiles between samples, we performed principal component analysis (PCA). Samples grown under the same conditions clustered together but separated by culture condition, and oxygen levels accounted for more than 90% of the variation in transcriptome profiles between samples (Fig. 1B). Interestingly, the *C. albicans* SC5314 cells cultured under aerobic conditions formed hyphae much more readily than those cultured under anaerobic conditions (Fig. 1C and D). Thus, *C. albicans* SC5314 cells grown in an anaerobic environment have a distinct morphology from those grown aerobically, and this is reflected in the downregulation of genes involved in hyphal transformation under anaerobic conditions (Table S3).

### Anoxia results in upregulation of genes associated with alternative respiration and stress adaptation processes in *C. albicans* SC5314

To identify the specific changes in gene expression in *C. albicans* SC5314 as a result of oxygen deprivation, we performed differential gene expression analysis and set aerobic expression as the baseline control. Genes that showed a twofold change (up or down) were considered differentially expressed, and a Benjamini-Hochberg adjusted *P*-value < 0.05 was used as the cut-off for statistical significance. A total of 2,181 genes met both criteria and were considered significantly differentially expressed between the two conditions. A total of 1,003 genes were identified as being significantly upregulated, and 1,178 genes were significantly downregulated relative to aerobically grown *C. albicans* SC5314. Of the top 50 upregulated genes, 21 were uncharacterized open reading frames (ORFs) but were predicted to encode mitochondrial proteins, adhesins, and endonucleases (Table S2).

**Fig 2:**
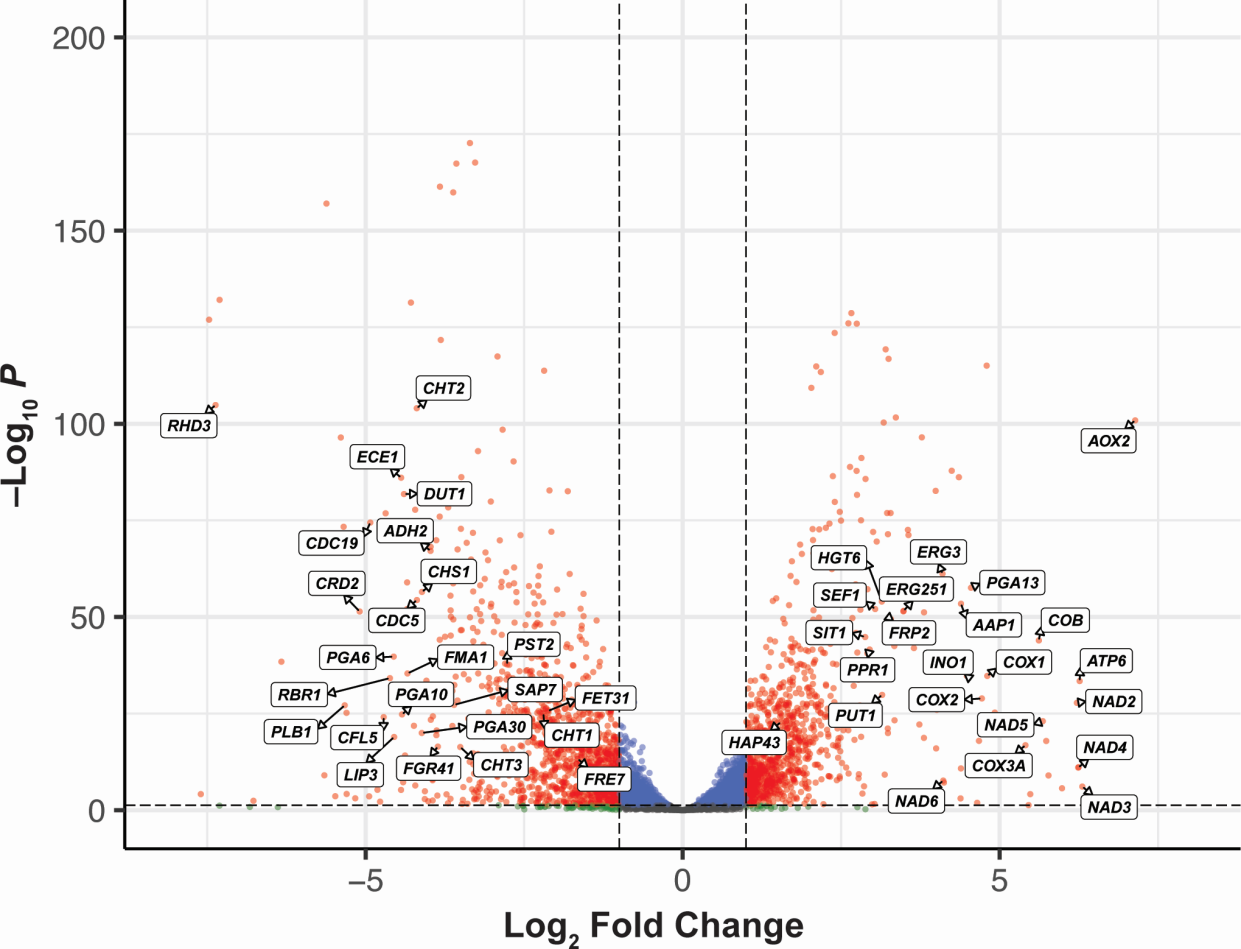
Significantly differentially expressed genes in the *C. albicans* SC5314 anaerobic cultures. Volcano plot showing the significantly differentially expressed genes in the anaerobic cultures relative to aerobic cultures. Genes that had a log_2_ fold change >1 (twofold increase) or <−1 (twofold decrease) and *P* < 0.05 were considered significant and are shown as red dots. Blue dots indicate genes that had a statistically significant change in expression (*P* < 0.05) but did not meet the log_2_ fold change cutoff. Green dots correspond to genes that did not reach statistical significance but did reach the threshold for log_2_ fold change, and gray dots indicate genes that did not meet either cutoff. An unlabeled version of this plot is included in Fig. S2.

**Fig 3:**
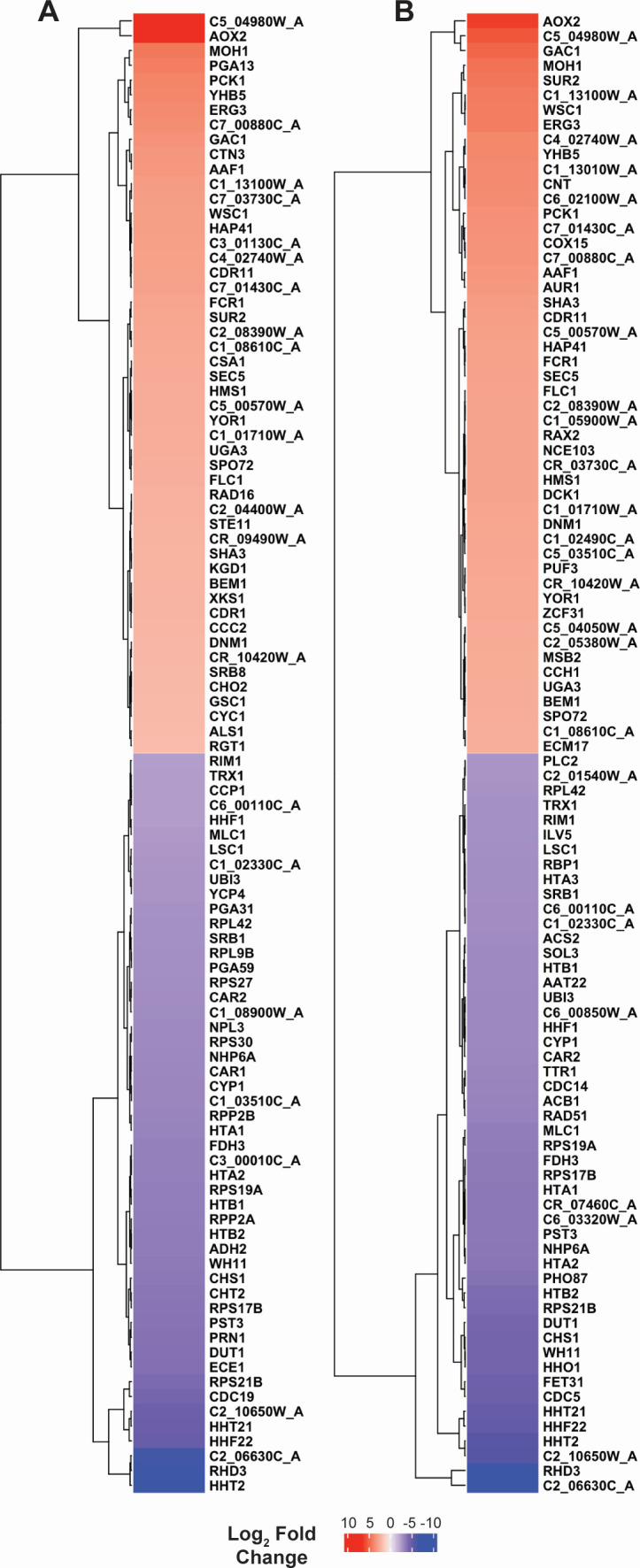
Heatmaps of the top 50 upregulated and downregulated genes in *C. albicans* strains under anaerobic vs aerobic conditions. (**A**) Differential gene expression in SC5314. (**B**) Differential gene expression in CHN1. Top differentially expressed genes were selected as >2 log_2_ fold change (positive or negative) and −log_10_ (adjusted *P*-value) >55. Data are shown as log_2_ fold change from aerobic expression. Red indicates higher gene expression under anaerobic conditions, and blue indicates lower gene expression under anaerobic conditions. Genes are grouped according to differential expression levels in each strain, with most upregulated at the top and most downregulated at the bottom.

**Fig 4:**
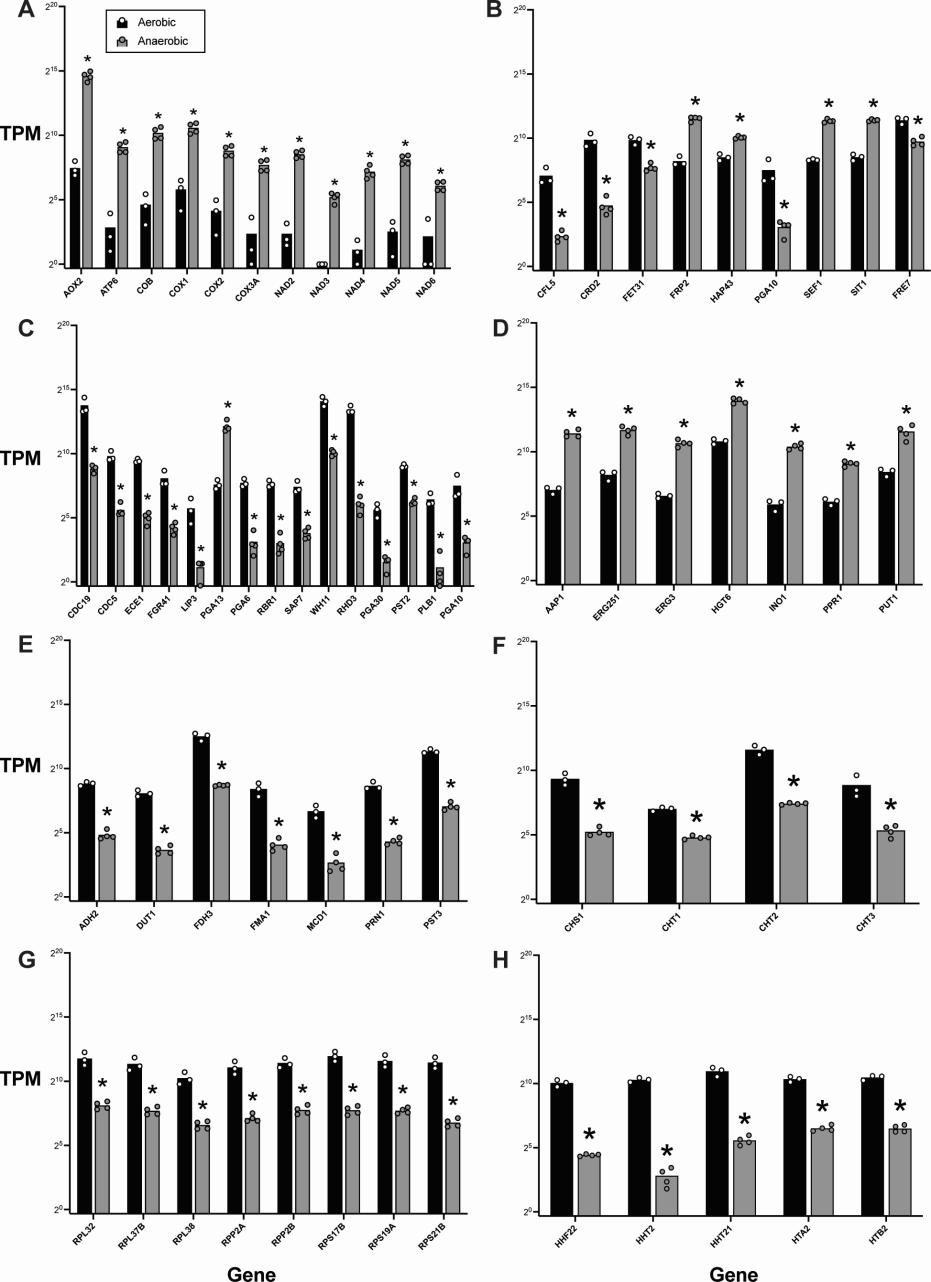
Normalized transcript counts for genes significantly affected by anaerobic growth in *C. albicans* SC5314. Transcripts per million (TPM) of genes involved in (**A**) respiration, (**B**) iron and copper homeostasis, (**C**) cell wall structure, filamentation, and virulence, (**D**) metabolism, (**E**) stress response, (**F**) chitin remodeling, (**G**) ribosome structure, and (**H**) histone structure under aerobic (black bars) and anaerobic (gray bars) conditions. Expression is shown on a log_2_ scale. Bars represent mean expression with individual points denoting TPM in a given culture. Data were obtained from three independent experiments. *P < 0.05 (anaerobic vs aerobic), unpaired *t*-test.

In our analysis of the top significantly upregulated genes under anaerobic conditions, the inducible alternative oxidase *AOX2* was one of the most strongly upregulated (Fig. 2 and 3A; Fig. S1A), with a 141-fold increase (log_2_ fold change = 7.140) in expression (Table S2). Only one uncharacterized ORF (C5_04,980W_A), encoding a putative adhesin-like protein, showed a greater increase in expression. Among the other top 20 most upregulated genes were subunits of the NADH-ubiquinone oxidoreductase (*NAD2*, *NAD3*, *NAD4*, *NAD5*, and *NAD6*), ATP synthase (*ATP6*), cytochrome c oxidase (*COX1*, *COX2*, and *COX3A*), and cytochrome b (*COB*; Fig. 4A). Overall, these results indicate that the absence of oxygen drives a major shift in the respiration pathways utilized by *C. albicans*, including activation of alternative respiration (i.e., non-electron transport chain [ETC]) by *AOX2* and upregulation of major mitochondrial enzyme complexes.

Alongside the observed changes in *C. albicans* SC5314 respiration following anaerobic growth, there was also a significant increase in the expression of a number of genes with fundamental roles in metabolism and maintenance of cellular structure. Several of these genes were associated with iron uptake, including the major regulators *HAP43* and *SEF1*, as well as *FRP2*, which encodes a ferric reductase-related protein involved in heme acquisition, and the siderophore transporter *SIT1* (Fig. 4B). The absence of oxygen also led to the induction of *PGA13* (encoding a glycosylphosphatidylinositol [GPI]-anchored protein that contributes to cell wall synthesis, acquisition of necessary surface properties, and filamentation; Fig. 4C). Evidence of metabolic changes included strong upregulation of *INO1*, *ERG3*, and *ERG251* (encoding enzymes that facilitate inositol and ergosterol biosynthesis) and several genes involved in amino acid sensing and metabolism (*AAP1*, *PUT1*, and *PPR1*; Fig. 4D). We also observed upregulation of *HGT6*—a putative glucose transporter also involved in the core stress response (Fig. 4E). Taken together, these data suggest that *C. albicans* SC5314 responds to oxygen deprivation by activating diverse pathways broadly associated with iron homeostasis, nutrient uptake, and cell wall biosynthesis.

### *C. albicans* SC5314 downregulates genes linked to pathogenicity, metal acquisition, and energy expenditure in response to anoxia

In our analysis of the downregulated genes in *C. albicans* SC5314 cultured under anaerobic conditions, we found a number of genes encoding histone and ribosomal subunits that had more than an eightfold decrease in expression (log_2_ fold change <−3; Fig. 2, 3A, 4G and H; Fig. S1A; Table S3). This coincided with a significant downregulation of several enzymes and regulators of metabolic pathways (*LIP3*, *DUT1*, *FMA1*, and *ADH2*; Fig. 4C and E). These data suggest an adaptive mechanism to conserve energy by reducing metabolic activity and protein synthesis when oxygen is no longer available.

In agreement with this notion, 9 out of the top 50 downregulated genes (which included 12 uncharacterized ORFs) are known to encode cell wall proteins or enzymes involved in cell wall synthesis (Fig. 2 and 3A; Fig. S1A; Table S3). These include structural proteins (*RHD3*, *PGA30*, and *PST2*), surface proteins involved in filamentation (*FGR41*, *RBR1*, and *CDC19*), and chitinases (*CHT1*, *CHT2*, *CHT3*, and *CHS1*; Fig. 4C and F). Notably, several of the most strongly downregulated genes under anaerobic conditions are known virulence factors: *ECE1* (candidalysin, a cytolytic peptide toxin essential for mucosal invasion), *PLB1* (a lysophospholipase used by *C. albicans* to damage and traverse host cell membranes), *CDC5* (a polo-like serine/threonine kinase involved in filamentation and virulence), and *SAP7* (a secreted aspartyl protease highly expressed during infection; Fig. 4C). Thus, these data are consistent with a model in which anaerobic growth under these conditions may promote *C. albicans* SC5314 commensalism by driving a decrease in expression of genes linked to pathogenicity, possibly as a consequence of reduced protein synthesis and ATP output.

While we had observed an upregulation of a number of genes associated with iron homeostasis (Fig. 2, 3A and 4B; Fig. S1A; Table S2), several genes implicated in iron and copper acquisition (*CRD2*, *CFL5*, *FET31*, and *FRE7*) were also significantly downregulated (Fig. 4B). Additionally, the iron-responsive GPI-anchored proteins *PGA6* and *PGA10* showed a large decrease in expression under anaerobic conditions relative to aerobic conditions (Fig. 4C). These results provide support for a model in which oxygen levels modulate metal uptake and homeostasis in *C. albicans* SC5314.

### *C. albicans* CHN1 shows more extensive oxygen-dependent differential gene expression than SC5314

**Fig 5:**
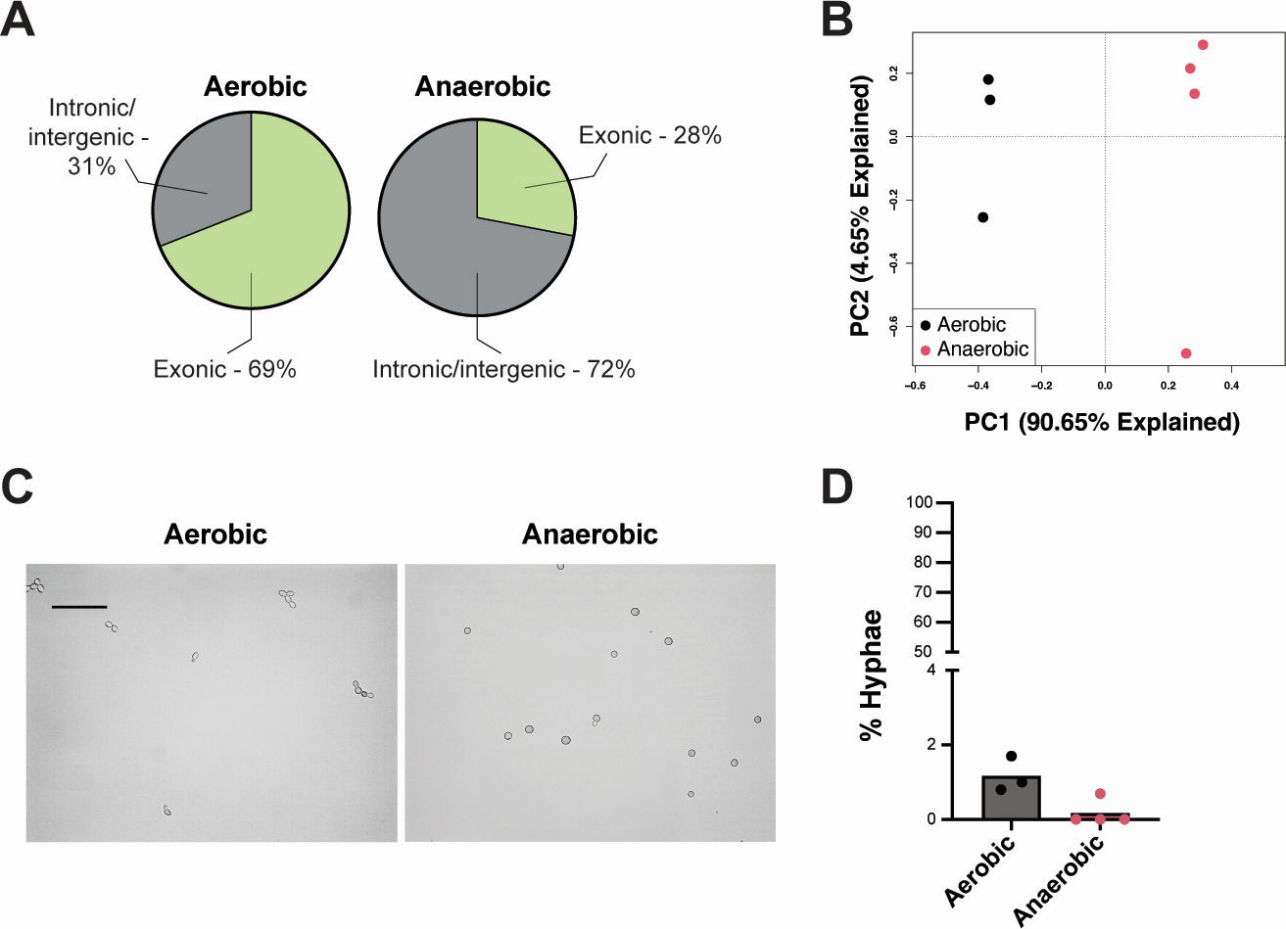
Sequencing results and phenotypic observations from *C. albicans* CHN1 cultures. (**A**) Percentages of sequenced reads mapped to exons (light green) vs intronic/intergenic regions (gray). (**B**) PCA of the aerobic and anaerobic cultures based on their transcriptomic profiles. (**C**) Brightfield micrographs of the aerobic and anaerobic cultures at the time of collection for RNA isolation. Scale bar = 20 µm. (**D**) Quantification of *C. albicans* hyphae in the aerobic and anaerobic cultures.

A number of studies have identified that *C. albicans* SC5314 differs from many other *C. albicans* strains in its filamentation capacity, colonization dynamics, and virulence (18–25). Thus, we compared the transcriptomic profile we identified in *C. albicans* SC5314 under anaerobic conditions to that of CHN1—a strain that has been used extensively by our laboratory (19, 23, 26, 27) and others (18, 20). The culture conditions, total RNA isolation, and RNA-seq protocols described earlier were repeated using *C. albicans* CHN1. For the aerobic cultures, we obtained an average of 2,106,523 reads per sample, of which >97% were successfully mapped to the *C. albicans* SC5314 reference genome (assembly 22). For the anaerobic cultures, an average of 32,019,275 reads were sequenced per sample, and >95% of those reads could be mapped to the reference genome. Sixty-nine percent of the total reads obtained from the CHN1 aerobic cultures and 28% of the reads from the anaerobic cultures mapped to exons (Fig. 5A). Overall, there were more genes identified as being significantly differentially expressed in *C. albicans* CHN1 (2,375) compared to *C. albicans* SC5314 (2,181) (Tables S4 and S5). Of those genes, 1,122 were significantly upregulated, and 1,253 were significantly downregulated in the *C. albicans* CHN1 anaerobic cultures compared to aerobic cultures. Similar to *C. albicans* SC5314, the differing oxygen levels accounted for >90% of the variation in transcriptome profiles between samples (Fig. 5B). However, in contrast to *C. albicans* SC5314 (Fig. 1C and D), *C. albicans* CHN1 cultures did not differ significantly in their morphology and were all comprised predominantly of yeast cells (Fig. 5C and D).

### *C. albicans* CHN1 and SC5314 exhibit similar differential gene expression patterns in response under anaerobic vs aerobic conditions

**Fig 6:**
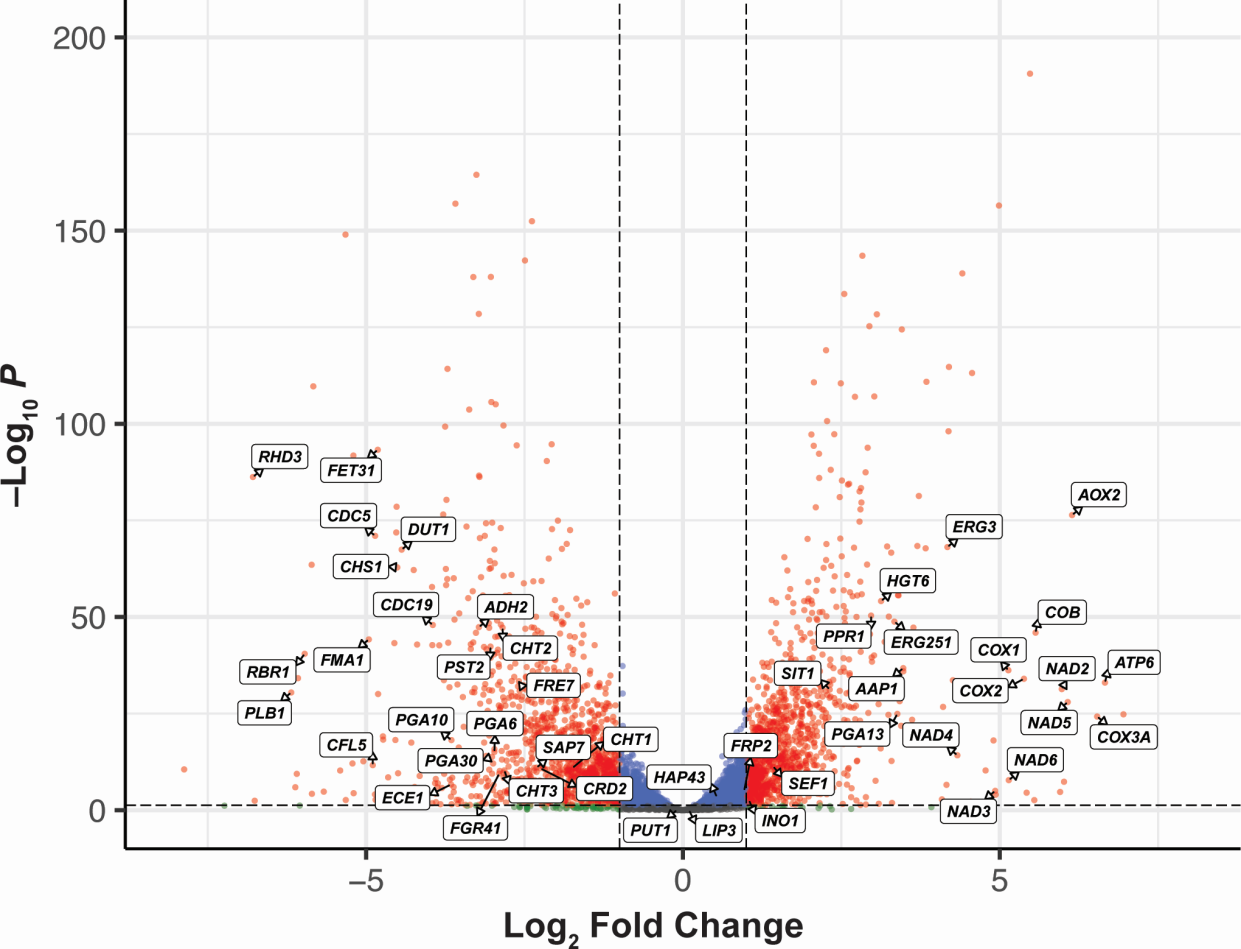
Significantly differentially expressed genes in the *C. albicans* CHN1 anaerobic cultures. Volcano plot showing the significantly differentially expressed genes in the anaerobic cultures relative to aerobic cultures. Genes that had a log_2_ fold change >1 (twofold increase) or <−1 (twofold decrease) and *P* < 0.05 were considered significant and are shown as red dots. Blue dots indicate genes that had a statistically significant change in expression (*P* < 0.05) but did not meet the log_2_ fold change cutoff. Green dots correspond to genes that did not reach statistical significance but did reach the threshold for log_2_ fold change, and gray dots indicate genes that did not meet either cutoff. An unlabeled version of this plot is included in Fig. S3.

**Fig 7:**
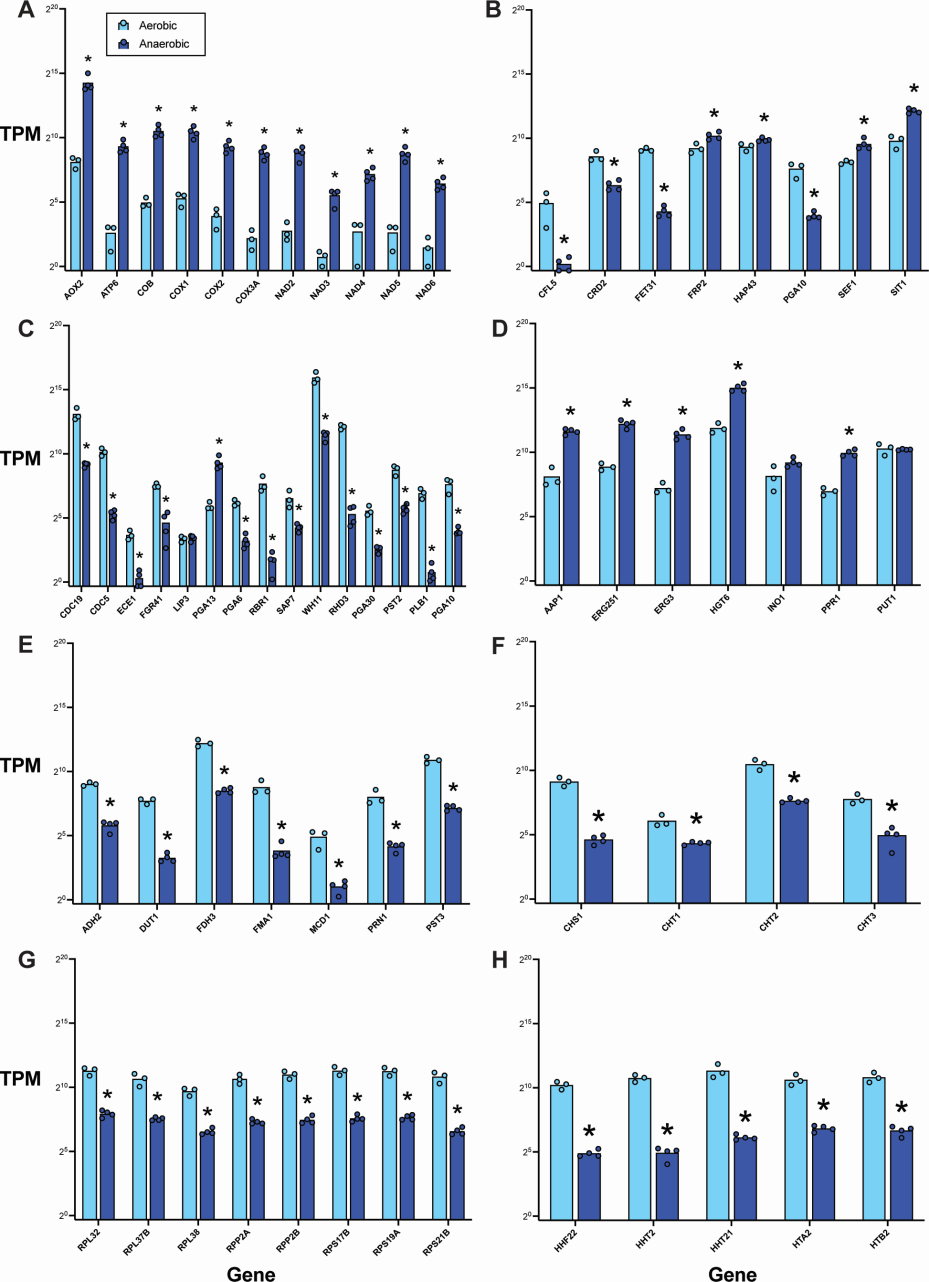
Normalized transcript counts for genes affected by anaerobic growth in *C. albicans* CHN1. Transcripts per million (TPM) of genes involved in (**A**) respiration, (**B**) iron and copper homeostasis, (**C**) cell wall structure, filamentation, and virulence, (**D**) metabolism, (**E**) stress response, (**F**) chitin remodeling, (**G**) ribosome structure, and (**H**) histone structure under aerobic (black bars) and anaerobic (gray bars) conditions. Expression is shown on a log_2_ scale. Bars represent mean expression with individual points denoting TPM in a given culture. Data were obtained from three independent experiments. *P < 0.05 (anaerobic vs aerobic), unpaired *t*-test.

Despite there being a greater number of genes that met both criteria for significant differential expression in *C. albicans* CHN1 compared to *C. albicans* SC5314, most of the top 50 upregulated genes under anaerobic conditions were consistent between the two strains (Fig. 3B and 6; Fig. S1B; Table S4). In particular, *AOX2* was one of the most strongly upregulated, though it had only a 71-fold increase in expression (log_2_ fold change = 6.142) compared to the 141-fold increase in *C. albicans* SC5314. Similar to *C. albicans* SC5314, subunits of the NADH-ubiquinone oxidoreductase, ATP synthase, cytochrome c oxidase, and cytochrome b were also among the top upregulated genes in *C. albicans* CHN1 (Fig. 7A). These findings indicate that anoxia also drives changes in the expression of major mitochondrial enzyme complexes and activates alternative respiration in *C. albicans* CHN1.

When we looked at the downregulated genes in *C. albicans* CHN1 under anaerobic conditions, we once again observed many parallels to *C. albicans* SC5314, including a strong decrease in expression of histones, ribosomal proteins, and cell wall proteins (Fig. 7C and E through H). Interestingly, there were fewer genes associated with filamentation and virulence represented among the top 50 most downregulated genes (Fig. 6; Table S5), suggesting that anoxia has a greater impact on these factors in *C. albicans* SC5314 than *C. albicans* CHN1. However, many of these genes (*ECE1*, *SAP7*, *FGR41*, *RBR1*, *CDC19*, *CHT2*, *CHT3*, *CHS1*, *CDC5*, and *PLB1*) were still significantly downregulated in *C. albicans* CHN1 (Fig. 7C), albeit to a lesser extent for some (Fig. 3; Fig. S1; Table S5). Thus, *C. albicans* CHN1 and *C. albicans* SC5314 undergo similar transcriptomic changes in response to oxygen deprivation under the growth conditions utilized in this study, indicating that these strains have comparable strategies for surviving in anoxia despite their genomic differences.

## DISCUSSION

In this study, we were interested in identifying molecular strategies used by *C. albicans* to adapt to extreme fluctuations in oxygen levels (i.e., normoxia vs anoxia), since the ability to withstand steep oxygen gradients is paramount to successful colonization and persistence throughout the gastrointestinal tract. We compared the transcriptomic profiles of *C. albicans* cultured in rich media at physiological temperature (37°C) under aerobic and anaerobic conditions. In our data analysis, we noted that a higher proportion of the reads in the aerobic cultures mapped to exons than reads in the anaerobic cultures (Fig. 1A and 4A). One explanation for this observation could be that the aerobic cultures are more transcriptionally active, as *C. albicans* grows and metabolizes at a significantly higher rate in the presence of oxygen (28). Alternatively, there might be increased RNA degradation in the anaerobic cultures or variations in alternative splicing between the two conditions; for example, stress responses in anaerobic cultures may result in more intron retention/exon skipping, while aerobic conditions enhance splicing efficiency (29). It is also entirely possible that these phenomena are occurring in combination to drive differences in exon mapping.

Differential gene expression analysis revealed a striking (141-fold) upregulation of the inducible alternative oxidase *AOX2* in the anaerobic cultures. This coincided with significant upregulation of genes encoding subunits of the mitochondrial complex I NADH-ubiquinone oxidoreductase (*NAD2*, *NAD3*, *NAD4*, *NAD5*, and *NAD6*) and complex V F_1_F_0_-ATP synthase (*ATP6* and *ATP8*). These findings indicate that when oxygen is not available to support respiration by the classical ETC pathway, *C. albicans* shifts to an alternative respiration pathway via induction of alternative oxidases (AOX). The roles of AOX and the alternative respiration pathway in *C. albicans* have been studied extensively under a variety of ETC-inhibitory conditions such as complex III inhibitors (e.g., antimycin A and KCN) (30), altered nutrient availability (30, 31), and oxidative stress (32, 33) but surprisingly not under fully anaerobic conditions. It was recently reported that maximal transactivation of *AOX2* following antimycin A treatment is mediated by the transcription factors Rtg1/Rtg3, Cwt1/Zcf11, and Zcf2 (30). However, only *ZCF2* expression was significantly increased in *C. albicans* under anaerobic conditions. The widespread downregulation of genes encoding histones and ribosomal protein subunits may be contributing to the repression of these transcription factors in our present study. Additionally, Rtg1 and Rtg3 were shown to be involved in the activation of *GAL* genes to convert galactose to glucose (34), so another possible explanation for their lack of induction could be the presence of glucose in the culture media. In summary, we have demonstrated that anoxia is an inducing stimulus for *AOX2* and alternative respiration in *C. albicans*, and the transcriptional networks regulating them are distinct from those reported for aerobic and hypoxic conditions.

A functional ETC is important not only for optimal *C. albicans* growth and metabolism but also for filamentation and virulence (30, 32, 35, 36). We found that *C. albicans* SC5314 readily undergoes hyphal morphogenesis when cultured in MRS at 37°C in ambient oxygen but assumes a predominantly yeast morphology in the absence of oxygen under otherwise identical conditions. The switch from ETC to alternative respiration and thus lower ATP production to fuel cellular functions likely explains why SC5314 does not filament as well under anaerobic conditions. Consistent with the reduced number of hyphae in the absence of oxygen, a number of genes known to be involved in *C. albicans* hyphal morphogenesis were significantly downregulated, as were many genes encoding virulence factors. The role of oxygen in *C. albicans* filamentation is further supported by studies showing that hypoxic conditions lead to a transcriptional response involving diminished expression of key morphogenetic and virulence-related genes, including those regulated by the transcription factors Efg1, Ahr1, and Tye7 (6–8, 14). Efg1 is a major regulator of hyphal morphogenesis and virulence that can act as either a repressor or inducer of filamentation under hypoxic conditions, depending on the pH and temperature within that niche (9). Of note, the early transcriptomic response of *C. albicans* to hypoxia can be mimicked by chemical inhibition of the ETC (8), providing further evidence for a link between oxygen availability, respiration (i.e., ATP production), and filamentation/virulence.

In addition to influencing overall morphology, oxygen levels can trigger changes in the relative abundance of specific *C. albicans* cell wall proteins (9, 10). Notably, hypoxia has been shown to induce β-glucan masking in SC5314 and several other isolates spanning four different *C. albicans* clades, which render them less susceptible to killing by phagocytes (3, 4). In this scenario, β-glucan exposure is modulated via a mechanism that involves both mitochondrial and cAMP-PKA signaling (4). We did not see significant changes in the expression of genes involved in the cAMP-PKA pathway (*CYR1*, *TPK1*, and *TPK2*) or mitochondrial signaling (*GOA1*), which were found to be critical for hypoxia-induced β-glucan masking, though there was a twofold decrease in the expression of the superoxide dismutase *SOD1* and a twofold increase in the expression of *UPC2* in the anaerobic cultures. There were also changes in the expression of genes involved in cell wall biosynthesis, including downregulation of chitinases and chitin synthases (*CHT1*, *CHT2*, *CHT3*, and *CHS1*), and upregulation of β-glucan glucanosyltransferases (*PHR3* and *PGA5*), glucosyltransferases (*ALG5*) and glucosidases (*EXG2* and *SCW11*; Tables S2 to S5). While we did not specifically measure levels of β-glucan, chitin, or mannan, these findings suggest that hypoxia and anoxia lead to distinct changes in cell wall architecture. Overall, rather than showing a clear shift toward a specific phenotypic state, this multifaceted transcriptomic response likely reflects a convoluted but flexible adaptation strategy that enables *C. albicans* to survive under suboptimal conditions.

Our analysis revealed significant changes in the expression of a number of genes associated with iron uptake and utilization under anaerobic conditions, including the upregulation of iron homeostasis regulators (*HAP43* and *SEF1*) and some iron uptake genes (*FRP2* and *SIT1*) and downregulation of other iron uptake genes (*FET31* and *FRE7*). In the gastrointestinal tract, *C. albicans* encounters varying and often abundant levels of iron (37), a crucial micronutrient for many cellular processes. However, excessive iron can be toxic as it leads to the formation of reactive oxygen species (ROS) that damage cellular components (38). In a recent study, high iron was reported to activate AOX in *C. albicans* as a means of protecting the fungus from ROS (31), indicating a possible association between the response to iron and oxygen fluctuations. Moreover, the transcriptional circuit controlling iron homeostasis (comprised of Sef1, Sfu1, and Hap43) is tightly linked to the balance between *C. albicans* commensalism and pathogenesis (10, 39–42), similar to the effects of oxygen. For example, in iron-limited niches like the bloodstream, Sef1, which activates Hap43 to increase iron uptake, is also required for hyphal growth and virulence in systemic infections (39, 41). In contrast, the gastrointestinal tract (where *C. albicans* typically exists as a commensal) constitutes an oxygen-replete, iron-rich environment in which Sfu1 represses iron utilization genes (39, 41). The results from our present study are the first to demonstrate a direct effect of oxygen on the transcriptional networks involved in iron uptake and homeostasis.

While comparisons of *C. albicans* strains often highlight their differences, our present study found that both SC5314 and CHN1 employ a similar adaptive strategy to oxygen deprivation. Most notably, anaerobically grown cells of both strains showed strong induction of *AOX2* and other genes associated with the alternative respiration pathway, along with downregulation of genes encoding histones, ribosomal proteins, and hyphal cell wall proteins. However, in contrast to SC5314, CHN1 did not readily undergo hyphal morphogenesis in either culture condition, despite being capable of hyphal invasion in the murine stomach (19). In a recent study, the repressor of filamentation Nrg1 was found to have divergent effects on *C. albicans* morphology and virulence in different strains (43). We did not observe differential expression of *NRG1* in either strain, although this does not rule out the possibility that Nrg1 interacts with different genetic circuits, thus evoking distinct biological responses to anoxia. Additional research is needed to identify the specific gene(s) that may be driving this difference between the two strains. In summary, our transcriptomic analysis has revealed that *C. albicans* SC5314 and CHN1 exhibit a coordinated response to anoxia characterized by the activation of alternative respiration through *AOX2* and a downregulation of genes linked to filamentation, virulence, and core cellular processes, highlighting a conserved adaptive mechanism across different strains.

## MATERIALS AND METHODS

### *C. albicans* culture conditions for transcriptomic analysis

*C. albicans* SC5314 and CHN1 cultures for each growth condition were prepared from overnight cultures grown to stationary phase in MRS broth at 37 °C. All overnight cultures were sub-cultured to a starting optical density at 600 nm (OD_600_) of 0.05, and the sub-cultures were grown to an exponential phase for RNA isolation. For the aerobic cultures, *C. albicans* SC5314 and CHN1 were sub-cultured into three separate flasks for each strain containing 20 mL MRS broth (Difco, Detroit, MI) and grown at 37 °C with gentle agitation (40 rpm) for 5–6 h. For the anaerobic cultures, overnight cultures of *C. albicans* SC5314 and CHN1 were prepared in an Anaerobe Systems AS-500 anaerobic chamber using MRS broth that had been left to reduce in the chamber for at least 24 h. Each strain was sub-cultured into four new flasks containing 10 mL reduced MRS broth and grown at 37 °C without agitation for 22 h. Oxygen concentration in the chamber was monitored continuously by an electronic sensor and verified with Mitsubishi RT Anaero-Indicator test strips. Micrographs from each culture were taken on a brightfield microscope at the time of cell harvest for RNA isolation (5–6 h for aerobic cultures and 22 h for anaerobic cultures) to assess cell morphology.

### *C. albicans* RNA isolation for transcriptomic analysis

Cells from each flask/growth condition were counted on a hemocytometer, then pelleted, and reconstituted in 1 mL sterile PBS. The reconstituted pellets were pelleted again and resuspended in 600 µL lysis buffer containing 1 M D-sorbitol (Sigma-Aldrich, St. Louis, MO) dissolved in sterile distilled water, 100 mM EDTA, and 14 mM β-mercaptoethanol. A total of 50 U zymolyase (Zymo Research, Irvine, CA) was added to each tube before incubating in a 37°C water bath for 1 h. Spheroplasts were harvested by centrifugation, then resuspended in Buffer RLT containing β-mercaptoethanol (Qiagen, Hilden, Germany), and vortexed to completely lyse the spheroplasts. RNA was purified using the RNeasy Mini Kit (Qiagen) according to the manufacturer’s instructions. RNA concentration and purity were evaluated using a nanodrop instrument (ThermoFisher, Waltham, MA) and Agilent Bioanalyzer (Agilent, Santa Clara, CA), respectively.

### Transcript sequencing

Library preparation and sequencing were performed at the Advanced Genomics Core at the University of Michigan. Briefly, samples underwent poly(A) enrichment using the NEBNext Poly(A) mRNA Magnetic Isolation Module (New England Biolabs, Ipswich, MA) followed by reverse transcription and library preparation using the UltraExpress RNA Library Prep Kit (New England Biolabs). Total RNA sequencing was performed on either an Illumina MiSeq or an Illumina NovaSeq to obtain 150 bp paired-end reads. The resulting sequences were uploaded to the Great Lakes computing cluster at the University of Michigan.

### Sequence processing and quantitation

Raw read quality was assessed using FastQC and MultiQC. Sequencing adaptors were identified and removed using Trimmomatic (v.39–2) and the TruSeq3 PE adaptor file, followed by quality checking again. Some samples showed low complexity at the 3′ end, suggesting remaining adaptors, which were subsequently removed using seqtk (https://github.com/lh3/seqtk). The trimmed sequences were quantified using Salmon v.1.8.0 by aligning the reads to the transcriptome of *C. albicans* strain SC5314 obtained from the Candida Genome Database (http://www.candidagenome.org). The Salmon index was constructed from the A22 build of *C. albicans* protein-coding sequences (C_albicans_SC5314_A22_current_default_coding.fasta). The resulting count files were downloaded and locally analyzed using R and R-studio. Gene annotations were obtained from FungiDB.org (44).

### Analysis of differentially expressed genes

Differential gene expression analysis was performed by importing the gene count tables into R using the tximport package to format them for analysis using DESeq2 (45). DESeq2 was run using a design model of design = ~organism + treatment + organism:treatment where organism consisted of two levels, SC5314 or CHN1, and treatment consisted of two levels, aerobic or anaerobic. The cutoff thresholds were set at >10 counts, and for differential expression, a *P*-value < 0.05 and a log_2_ fold change <−1 or >1. Visualization of the results was performed in R using the following packages: tidyverse, ggplot2, vegan, ggvenn, EnhancedVolcano, and ComplexHeatmap. Pathway analysis of significant differentially regulated genes was performed on FungiDB.org, limiting results to SC5314 genes.

### Statistical analysis

Differentially expressed genes were identified using the negative binomial distribution model in the DESeq2 R package, which applies the Wald test to compare gene expression between groups and incorporates size factors to normalize counts across samples. Adjusted *P*-values were obtained using the Benjamini-Hochberg procedure (45). An unpaired *t*-test was used for comparisons of the percent hyphae in aerobic and anaerobic cultures. Multiple unpaired *t*-tests with a two-stage step-up (Benjamini, Krieger, and Yekutieli) to correct for multiple comparisons were used to identify statistically significant differences in transcripts per million values between the aerobic and anaerobic cultures.

## Data Availability

All sequencing data in this study are available in the NCBI SRA under the BioProject accession number PRJNA1142969, which can be found at https://www.ncbi.nlm.nih.gov/sra/PRJNA1142969.

## SUPPLEMENTAL MATERIAL

Supplemental figures spectrum.03024-24-s0001.pdf Fig. S1 to S3.

Supplemental figure legends spectrum.03024-24-s0002.docx Legends for Fig. S1 to S3.

Table S1 spectrum.03024-24-s0003.xlsx Sequencing run information and read mapping statistics.

Table S2 spectrum.03024-24-s0004.xlsx Significantly upregulated genes in *C. albicans* SC5314 under anaerobic vs aerobic conditions.

Table S3 spectrum.03024-24-s0005.xlsx Significantly downregulated genes in *C. albicans* SC5314 under anaerobic vs aerobic conditions.

Table S4 spectrum.03024-24-s0006.xlsx Significantly upregulated genes in *C. albicans* CHN1 under anaerobic vs aerobic conditions.

Table S5 spectrum.03024-24-s0007.xlsx Significantly downregulated genes in *C. albicans* CHN1 under anaerobic vs aerobic conditions.

ASM does not own the copyrights to Supplemental Material that may be linked to, or accessed through, an article. The authors have granted ASM a non-exclusive, world-wide license to publish the Supplemental Material files. Please contact the corresponding author directly for reuse.
