# Supplementary material for: Transcriptomic insights into Candida albicans adaptation to an anaerobic environment
Source: Microbiol Spectr. 2025 May 22;13(7):e03024-24. doi: 10.1128/spectrum.03024-24 (PMC12210919; doi:10.1128/spectrum.03024-24)
Supplement: Supplemental figure legends — Legends for Fig. S1 to S3. [file spectrum.03024-24-s0002.docx]

**SUPPLEMENTAL FIGURE LEGENDS**

**Fig. S1: Heatmaps of differentially expressed genes of interest in *C. albicans* strains under anaerobic vs. aerobic conditions.** (**A**) Differential gene expression in SC5314. (**B**) Differential gene expression in CHN1. Data is shown as log_2_ fold change from aerobic expression. Red indicates higher gene expression under anaerobic conditions, and blue indicates lower gene expression under anaerobic conditions. Genes are listed according to differential expression levels in each strain, with most upregulated at the top and most downregulated at the bottom.

**Fig. S2: Unlabeled volcano plot of the significantly differentially expressed genes in the SC5314 anaerobic cultures relative to aerobic cultures.** Genes that had a log_2_ fold change > 1 (2-fold increase) or < -1 (2-fold decrease) and P < 0.05 were considered significant and are shown as red dots. Blue dots indicate genes that had a statistically significant change in expression (P < 0.05) but did not meet the log_2_ fold change cutoff. Green dots correspond to genes that did not reach statistical significance but did reach the threshold for log_2_ fold change. Gray dots indicate genes that did not meet either cutoff.

**Fig. S3: Unlabeled volcano plot of the significantly differentially expressed genes in the CHN1 anaerobic cultures relative to aerobic cultures.** Genes that had a log_2_ fold change > 1 (2-fold increase) or < -1 (2-fold decrease) and P < 0.05 were considered significant and are shown as red dots. Blue dots indicate genes that had a statistically significant change in expression (P < 0.05) but did not meet the log_2_ fold change cutoff. Green dots correspond to genes that did not reach statistical significance but did reach the threshold for log_2_ fold change. Gray dots indicate genes that did not meet either cutoff.
